# Künstliche Intelligenz und akute Nierenschädigung

**DOI:** 10.1007/s00063-024-01111-5

**Published:** 2024-02-23

**Authors:** Fabian Perschinka, Andreas Peer, Michael Joannidis

**Affiliations:** Gemeinsame Einrichtung für Internistische Notfall- und Intensivmedizin, Department Innere Medizin, Medizinische Universität Innsbruck, Anichstraße 35, 6020 Innsbruck, Österreich

**Keywords:** Maschinelles Lernen, Algorithmen, Nierenversagen, Prädiktion, AKI Phänotypen, Machine learning, Algorithms, Kidney failure, Forecasting, AKI Phenotypes

## Abstract

Die Digitalisierung hält zunehmend Einzug auf den Intensivstationen und mit ihr die künstliche Intelligenz (KI) bei kritisch kranken Patient*innen. Ein vielversprechendes Gebiet für den Einsatz von KI liegt im Bereich der akuten Nierenschädigung (AKI). Hierbei beschäftigt sich KI derzeit noch vorwiegend mit der Prädiktion von AKI und vereinzelt mit der Klassifizierung bestehender AKI in verschiedene Phänotypen. In der Prädiktion kommen unterschiedliche KI-Modelle zum Einsatz. Die hiermit erreichten „Area-under-the-receiver-operating-characteristic-curve“-Werte (AUROC-WERTE) divergieren stark und werden von diversen Faktoren, wie dem Vorhersagezeitraum und der AKI Definition, beeinflusst. Die meisten Modelle weisen eine AUROC zwischen 0,650 und 0,900 auf, wobei bei Vorhersagen weiter in die Zukunft und dem Anwenden der „Acute-kidney-injury-network“-Kriterien (AKIN-Kriterien) niedrigere Werte vorliegen. Der Phänotypisierung gelingt es zwar bereits, Patient*innen in Gruppen mit unterschiedlichem Risiko für erhöhte Sterblichkeit oder Bedarf einer Nierenersatztherapie (RRT) einzuteilen, jedoch fehlen noch daraus abgeleitete Ätiologien und therapeutische Konsequenzen. All den unterschiedlichen Modellen liegen allerdings KI-spezifische Schwächen zugrunde. Der Einsatz von großen Datenbanken ermöglicht es nicht, zeitnah rezente Veränderungen in der Therapie und die Implementierung neuer Biomarker in einem aussagekräftigen Anteil zu enthalten. Aus diesem Grund dominieren Serumkreatinin und Harnzeitvolumen die aktuellen KI-Modelle und führen mit den bekannten Limitationen zu einer Begrenzung der Performance der derzeitigen Modelle. Die immer komplexer werdenden Modelle ermöglichen es den Ärzt*innen nicht mehr nachzuvollziehen, auf welcher Grundlage die Warnung eines bevorstehenden AKI errechnet wird und nachfolgend eine Therapieinitiierung stattfinden soll. Der erfolgreiche Einsatz von KI in der klinischen Routine wird maßgeblich vom Vertrauen der behandelnden Ärzt*innen in die Systeme und dem Überwinden der bereits genannten Schwächen geprägt sein. Als entscheidende Instanz wird der Kliniker/die Klinikerin bei kritisch kranken Patient*innen durch das Vereinen von messbaren mit nichtmessbaren Parametern allerdings unersetzlich bleiben.

## Abstract (en)

Digitalization is increasingly finding its way into intensive care units and with it artificial intelligence (AI) for critically ill patients. One promising area for the use of AI is in the field of acute kidney injury (AKI). The use of AI is primarily focused on the prediction of AKI, but further approaches are also being used to classify existing AKI into different phenotypes. Different AI models are used for prediction. The area under the receiver operating characteristic curve values (AUROC) achieved with these models vary and are influenced by several factors, such as the prediction time and the definition of AKI. Most models have an AUROC between 0.650 and 0.900, with lower values for predictions further into the future and when applying Acute Kidney Injury Network (AKIN) instead of KDIGO criteria. Classification into phenotypes already makes it possible to categorize patients into groups with different risks of mortality or requirement of renal replacement therapy (RRT), but the etiologies or therapeutic consequences derived from this are still lacking. However, all the models suffer from AI-specific shortcomings. The use of large databases does not make it possible to promptly include recent changes in therapy and the implementation of new biomarkers in a relevant proportion. For this reason, serum creatinine and urinary output, with their known limitations, dominate current AI models for prediction impairing the performance of the current models. On the other hand, the increasingly complex models no longer allow physicians to understand the basis on which the warning of a threatening AKI is calculated and subsequent initiation of therapy should take place. The successful use of AIs in routine clinical practice will be highly determined by the trust of the physicians in the systems and overcoming the aforementioned weaknesses. However, the clinician will remain irreplaceable as the decisive authority for critically ill patients by combining measurable and nonmeasurable parameters.

## Hintergrund

Die akute Nierenschädigung (AKI) zählt zu den häufigsten Komplikationen bei hospitalisierten (12,2 %; [1]) sowie kritisch kranken Patient*innen (57,3 %; [2]) und ist mit einer erhöhten Morbidität und Mortalität assoziiert [3]. Die hohe Inzidenz dieses Syndroms und seine negativen Folgen machen es zu einem attraktiven Feld, um mit neuen Ansätzen das Auftreten von AKI vorherzusagen, besser zu charakterisieren und im besten Fall zu verhindern. Rasche Entwicklungen auf dem Gebiet der künstliche Intelligenz (KI; „artificial intelligence“, AI) lassen den Einsatz dieser Technologie vielversprechend erscheinen, um Kliniker*innen im Alltag zu unterstützen. Wenn man berücksicht, dass das derzeitig empfohlene KDIGO Bündel zur AKI Prävention nicht einmal bei Hochrisikopatienten eingehalten wird, so besteht die Hoffnung, dass bei frühzeitiger AKI Prädiktion durch KI diese universalen Präventionsmaßnahmen konsequenter zum Einsatz kommen werden [4].

Um die Stärken und Schwächen der KI besser zu verstehen, ist es notwendig, sich mit den verschiedenen KI-Modellen auseinanderzusetzen und ihr Potenzial auf dem Gebiet der AKI-Forschung realistisch einzuschätzen. Dieser Review soll ein differenziertes Bild auf die Möglichkeiten der KI werfen, eine AKI frühzeitig zu erkennen, Phänotypen zu klassifizieren und letztendlich Therapieentscheidungen zu optimieren.

## Funktionsweise von künstlicher Intelligenz

**Figure 1:**
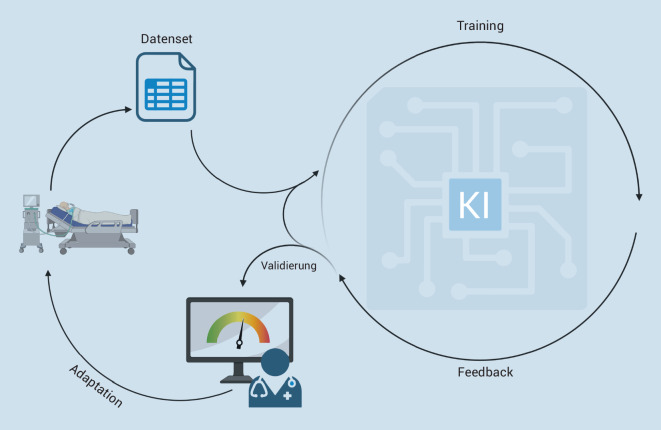

Im Gegensatz zur klassischen Programmierung, die auf festen Regeln (meist Algorithmen basierend auf logistischer Regression) zurückgreift, um eine Lösung zu ermitteln, werden bei KI lediglich Rahmenbedingungen für die Lösung von Problemen vorgegeben. Die KI-Systeme „lernen“ anhand von zahlreichen Wiederholungen mit entsprechendem Feedback (Training), wie eine vorgegebene Lösung (z. B. Diagnose, Prognose eines Krankheitsbilds) besser erreicht werden kann und somit das Ergebnis genauer und die Verfahren effizienter werden (Abb. 1).

Die KI-Systeme „lernen“ anhand von zahlreichen Wiederholungen mit entsprechendem Feedback

Für diese Verfahren wird daher generell die Bezeichnung „machine learning“ (ML) favorisiert. Demgegenüber stehen neuronale Netzwerke, die, wenn die Anzahl der Schichten erhöht wird, als „deep learning“ (DL) bezeichnet werden.

Um KI-Modelle für die Prädiktion oder Klassifizierung von AKI zu trainieren, werden große Datensätze mit verschiedenen Krankheitsverläufen verwendet. Patient*innen werden hierbei anhand vordefinierter Kriterien in „AKI aufgetreten“ und „AKI nicht aufgetreten“ kategorisiert. Die bei KI zur Anwendung kommenden Verfahren sind also entweder ML oder DL.

## Machine-learning-Modelle

Um die Therapie einer Erkrankung (z. B. AKI) frühzeitig adaptieren zu können oder ein Auftreten gänzlich zu verhindern, ist es entscheidend, möglichst früh Patient*innen mit einem hohen Risiko zu erkennen. Während sich für kurzzeitige Vorhersagen die logistische Regression etabliert hat, wird bei großen Datenmengen und komplexen Analysen zunehmend die Verwendung von KI empfohlen [5]. Jeder der KI-Algorithmen weist eine Sensitivität und Spezifität auf. Grafisch wird dies in ein Diagramm mit x‑Achse (1-Spezifität) und y‑Achse (Sensitivität) aufgetragen und die „area under the receiver operating characteristic curve“ (AUROC) abgebildet, um die Leistungsfähigkeit zu bestimmen. Je höher die AUROC ist, desto besser unterscheidet das Modell zwischen „AKI“ und „kein AKI“, wobei Werte unter 0,7 üblicherweise als wenig aussagekräftig und Werte über 0,9 als ausgezeichnet eingestuft werden.

**Figure 2:**
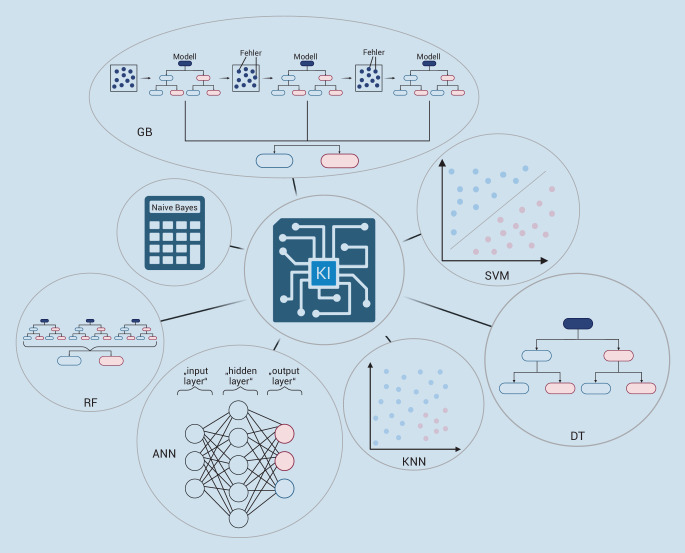

Zur Prädiktion von AKI wurden bislang unterschiedlich funktionierende KI-Algorithmen verwendet ([6]; Abb. 2):

### K-nearest-neighbour-Algorithmus

Der K‑nearest-neighbour-Algorithmus (KNN) zeichnet sich durch seine Einfachheit aus und ist in der Lage, Daten nach Kategorien (Diagnosen) zu klassifizieren. Die zu untersuchenden Daten/Patient*innen werden anhand des gelernten Datensets in einem Diagramm platziert. Abhängig von den Nachbarn in dem Diagramm werden die Daten z. B. in „AKI“ und „kein AKI“ eingeteilt. Die Zahl K bestimmt, wie viele Nachbarn hierfür herangezogen werden [7].

### „Support vector machine“

Die „support vector machines“ (SVM) bilden in einem Koordinatensystem eine Grenze zwischen den beiden einzuteilenden Klassen. Diese Grenze wird „hyperplane“ genannt. Im Lernprozess wird ein Modell (Gewichtung der Parameter) entwickelt, sodass die untersuchten Daten/Patient*innen im größtmöglichen Abstand zur „hyperplane“ auf der richtigen Klassifizierungsseite angeordnet werden. Da dieses System eine große Rechenleistung benötigt, jedoch nicht durch die Anzahl der Parameter beeinflusst wird, eignet es sich besonders für kleine Samples mit vielen Parametern [8].

### „Decision tree“

Der Decision-tree(DT)-Algorithmus besteht aus Abfolgen von Entscheidungen („decision trees“), die einen Grenzwert haben, ab welchem die eine oder die andere Abzweigung gewählt wird. Die DT-Algorithmen entwickeln in den Trainings eine Abfolge von Entscheidungen, die es ermöglichen, die zu untersuchenden Daten/Patient*innen mit der geringsten Fehlerrate zu klassifizieren. Auf jeder Ebene des Entscheidungsbaums wird die Grenze der Entscheidung gesucht, die den größten Effekt auf die Zuteilung hat. Dies wird solange wiederholt, bis alle Datenpunkte zugeordnet sind. Der Vorteil der „decision trees“ ist die vergleichsweise nachvollziehbare Zuordnung [9].

### „Random forest“

Der Random-forest(RF)-Algorithmus basiert auf randomisierten „decision trees“. Jeder dieser „trees“ klassifiziert einzeln und die Mehrheit dieser Einteilungen entscheidet über die Gesamteinteilung. Der „random forest“ eignet sich vor allem für große Datensätze und verhindert eine Überanpassung der Entscheidungen. Mit der Anzahl der „decision trees“ wird die Balance zwischen Effizienz und Genauigkeit gesteuert [10, 11].

### „Gradient boosting“ und „extreme gradient boost“

Ein Gradient-boosting(GB)-Algorithmus kombiniert viele Modelle mit multiplen „decision trees“ und einer geringen Genauigkeit zu einem Modell mit hoher Genauigkeit. Dieses zusammengesetzte Modell führt zu einer finalen Klassifizierung [12, 13].

### „Naive Bayes“

Das Naive-Bayes(NB)-Modell beruht auf dem bekannten Bayes-Theorem. Hierbei werden, je nach der Häufigkeit des Auftretens eines Werts, verschiedene Wahrscheinlichkeiten in dem Datenset multipliziert. Trotz der Ungenauigkeit der Annahme, dass jeder Parameter unabhängig von der Klassifizierung ist, gilt es als ein valides Instrument zur Einteilung [14–16].

### Deep-learning-Modelle: „artificial neural network“

„Artificial neural networks“ (ANN) sind die komplexesten Verfahren, die zur Prädiktion von AKI regelmäßig verwendet werden. Sie bestehen aus vielen „Neuronen“, die in verschiedenen Lagen („input-“, „hidden-“ und „output-layer“) angeordnet sind. Ein „Neuron“ enthält mathematische Funktionen. In der „hidden-layer“ nimmt das Neuron die Informationen der anderen Neuronen auf, gewichtet diese Inputs, rechnet die Ergebnisse und gibt die Informationen an Neuronen in derselben Lage oder einer Lage darunter weiter [17]. Die unterschiedlichen Gewichtungen werden anhand von Trainingsmodellen so lange adaptiert, bis die Ergebnisse der vorgegebenen Lösung entsprechen und anschließend bei realen Daten bzw. Patient*innen zum Einsatz kommen.

## Prädiktion einer akuten Nierenschädigung

Erste Ergebnisse von auf DL basierten KI-Modellen zur Vorhersage von AKI bei kritisch kranken Patient*innen im Jahr 2017 waren bereits vielversprechend, da sie eine ähnlich gute Vorhersagekraft wie der renale Biomarker Neutrophilen-Gelatinase-assoziiertes Lipocalin (NGAL, bestimmt im Serum) aufwiesen [18]. Um die Modelle für den klinischen Einsatz zu verbessern, wurden diese unter verschiedensten Voraussetzungen getestet, wodurch sich die Studienlage hinsichtlich der Performance von KI-Technologien zur Prädiktion von AKI im letzten Jahrzehnt exponentiell entwickelt hat. Bei Betrachtung der Ergebnisse müssen jedoch die exakten Bedingungen der Verfahren berücksichtigt werden.

Einen wichtigen Faktor in den Analysen stellen die jeweils verwendeten Datenbanken dar

Einen wichtigen Faktor in den Analysen stellen die jeweils verwendeten Datenbanken in der Trainingsphase und zur Validierung dar. Viele Untersuchungen verwendeten die etablierte Datenbank „medical information mart for intensive care“ (MIMIC) III, wobei ein K‑nearest-neighbour-Modell eine vergleichsweise schlechte Performance (AUROC: 0,664; [19]) aufwies, während ein „support vector machine“-Modell mehr überzeugte (AUROC: 0,861; [20]). Bei einer Analyse mit einer anderen Datenbank (DECLARE) kam ein „support vector machine“-Modell auf einen niedrigeren, aber vergleichbaren Wert (AUROC: 0,82; [21]). Zudem ist der Einfluss der berücksichtigten Parameter wichtig: Oftmals werden nur die Standardmarker (Kreatinin, Harnausscheidung, Risikofaktoren) mit den bekannten Limitation berücksichtigt.

Zudem scheint die Definition der AKI einen großen Einfluss auf das Ergebnis zu haben und ein relevanter Faktor für die Heterogenität der Ergebnisse zu sein [6, 22]. Abhängig davon, ob die Kidney-Disease-Improving-Global-Outcomes-Kriterien (KDIGO-Kriterien) [23] oder die Acute-kidney-injury-network-Kriterien (AKIN-Kriterien) [24] zum Einsatz kamen, zeigten sich unterschiedliche Werte für Sensitivität und Spezifität. Allgemein lässt sich jedoch feststellen, dass die Ergebnisse zur Prädiktion der AKI nach den KDIGO-Kriterien eine höhere Genauigkeit aufweisen als nach den AKIN-Kriterien (Tab. 1).

**Table 1:** 

*AKI (nach Kidney Disease *–* Improving Global Outcomes, KDIGO) während des Aufenthalts*	*AUROC*
„Logistic regression“	0,686–0,930
„Naive Bayes“	0,687–0,819
„Support vector machine“	0,720–0,900
„Decision trees“	0,637–0,781
„Generalized additive model“	0,858
„Random forest“	0,709–0,911
„Adaptive boost M1“	0,751
„Gradient boosting“	0,741–0,900
„Nearest neighbour“	0,664–0,920
„Artificial neural network“	0,720–0,921
*AKI (nach „acute kidney injury network“, AKIN) während des Aufenthalts*	*AUROC*
„Logistic regression“	0,660
„Naive Bayes“	0,654
„Support vector machine“	0,621
„Decision trees“	0,639
„Generalized additive model“	0,777
„Gradient boosting“	0,752

Die Tab. 1 gibt einen Überblick über die verschiedenen AUROC abhängig von dem verwendeten ML-Algorithmus und der AKI-Definition [19–21, 25–37].

Ein wesentlicher Faktor für die Verlässlichkeit einer AKI-Prädiktion ist der Vorhersagezeitraum, also wie weit in die Zukunft versucht wird, eine Vorhersage zu treffen. Erreichen manche Modelle für das KDIGO-Stadium II binnen 24 h eine sehr gute Prädiktion mit AUROC-Werten von 0,900, sinkt deren Vorhersagekraft mit zunehmender Dauer (z. B. für 48 h AUROC = 0,870). Eine Ausweitung des Prognoseintervalls auf 5 Tage führte zu einer weiteren erheblichen Abnahme der Verlässlichkeit der Modelle (z. B. AUROC von nur mehr 0,670). Selbst komplexe Analysen mit neuralen Netzwerken zeigten zwar in dem Vorhersagezeitraum von maximal 48 h eine ausgezeichnete AUROC (0,921), jedoch nahm auch hier die Genauigkeit mit zunehmendem Prognoseintervall (9 Tage) erheblich ab (0,720; [28]).

In einer Metaanalyse zeigte sich kein Unterschied in der Performance der untersuchten ML-Modelle

In einer Metaanalyse zur AKI-Prädiktion mit 24 Studien und insgesamt 82 verschiedenen ML-Modellen zeigte sich kein Unterschied in der Performance zwischen den ML-Modellen („random forest, artificial neural network, support vector machine, Bayes network“ und „gradient boosting“) und der logistischen Regression hinsichtlich der mittleren AUROC. Bei Betrachtung der KI-Methoden untereinander waren Gradient-boosting-Modelle („gradient boosting“ und „extreme gradient boosting“) signifikant besser als „artificial neural network, support vector machine“ und „Bayes network“, jedoch konnte im Vergleich mit Random-forest-Modellen kein signifikanter Unterschied beobachtet werden. Zusätzlich zeigte die Analyse, dass die Modelle der meisten Studien Kreatinin (im Blut oder Urin) zur Prädiktion heranzogen [38].

Eine Metaanalyse zur Prädiktion von AKI im perioperativen Bereich untersuchte insgesamt 19 Studien mit mehr als 300.000 Patienten und errechnete eine gute AUROC von 0,83 (95 %-Konfidenzintervall: 0,80–0,86). Bei Annahme einer 50 %igen Wahrscheinlichkeit für ein postoperatives AKI liegt bei einem positiven Testergebnis somit zu 76 % ein AKI vor, während bei einem negativen Testergebnis das Risiko bei 23 % liegt. Obwohl die Robustheit der prädiktiven Vorhersage hervorgehoben wird, ist der mögliche Bias in der Analyse beachtenswert. Das Risiko eines Bias in den Studien wurde als insgesamt hoch angegeben, wobei hier der größte Teil durch die Analysemethoden bedingt ist. Die Studienautoren mutmaßen zudem, dass bereits ein Plateau in der Entwicklung auf Basis der derzeit verwendeten Parameter erreicht sein könnte [6].

Im Vergleich zur ärztlichen Einschätzung zeigte die ML-basierte KI eine bessere Präzision zum Zeitpunkt der Aufnahme, allerdings glichen sich nach 24 h beide Einschätzungen in ihrer Genauigkeit an [29]. Durch die Miteinbeziehung zusätzlicher neuer Biomarker für eine Nierenschädigung könnte die Prognose deutlich verbessert werden. Ein K‑nearest-neighbour-Modell kam unter Einbeziehung von NGAL auf eine sehr gute Prädiktion (AUROC: 0,92; [30]). Obwohl inzwischen einige neue Biomarker, wie Cystatin C, „kidney injury molecule“ (KIM) 1, NGAL und „tissue inhibitor of metalloprokinase“ (TIMP) 2/„insulin-like growth factor binding protein“ (IGFBP) 7, für die Früherkennung der AKI untersucht werden [18, 39, 40], fehlen bislang ausreichend große Datensätze mit diesen Parametern, um sie in ML-Modellen zu untersuchen.

Das Risiko eines Bias in den Studien wurde als insgesamt hoch angegeben

Ein erster vielversprechender Ansatz mithilfe von DL stellt die Studie von Tomasev et al. dar. Auf Basis eines longitudinalen Datensatzes von 703.782 erwachsenen Patienten (sowohl ambulante als auch stationäre Daten der Veterans Administration) ließen sich 56 % der stationären AKI-Episoden und 90 % aller Episoden mit Nierenersatztherapie mit einer Vorlaufzeit bis zu 48 h vorhersagen. Das retrospektive Design und die Tatsache, dass 94 % der Patient*innen Männer waren, limitiert bislang die Generalisierbarkeit dieses Ansatzes.

## „Electronic alerts“ bei drohender akute Nierenschädigung

**Figure 3:**
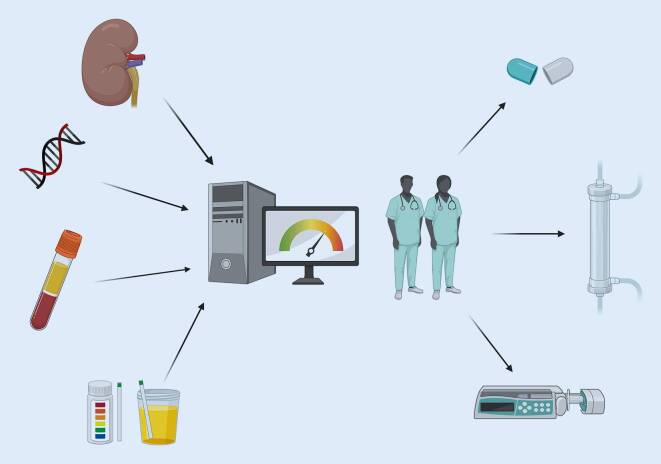

Alle Modelle zur Prädiktion errechnen eine Wahrscheinlichkeit für das Auftreten von AKI und sollen den Kliniker*innen durch Warnungen („electronic alerts“, E‑Alerts) unterstützen und zu früheren Gegenmaßnahmen führen. Der Nutzen dieser Warnsysteme ist bislang jedoch umstritten. Untersuchungen von E‑Alerts basierend auf Veränderungen im Serumkreatinin bzw. Harnzeitvolumen an über 10.000 eingeschlossenen Patient*innen zeigten weder eine reduzierte Mortalität noch eine seltenere Notwendigkeit zur Initiierung von Nierenersatztherapien. Auch Änderungen im Volumenmanagement konnten nicht beobachtet werden [41]. Demgegenüber stehen Untersuchungen, die durch gesteigerte „Awareness“ ein vermehrtes Absetzen von nephrotoxischen Medikamenten nachweisen konnten, ohne das anschließende Outcome zu untersuchen [42]. In fixer Kombination mit Maßnahmenbündeln (z. B. Audits, ABCDE-IT; Abb. 3) wurde jedoch ein positiver Einfluss auf die Mortalität berichtet. Das ABCDE-IT Bündel enthält Überlegungen und Interventionen, die beim Auftreten von AKI durchgeführt werden sollten (A „acute complications“, B „blood pressure“, C „catheterize“, D „drugs“, E „exclude obstruction“, I „investigations“, T „treat cause”) [43, 44]. Einschränkend ist festzuhalten, dass die bisherigen Untersuchungen zu E‑Alerts auf simplen Algorithmen beruhten, bei denen lediglich kontinuierlich der Verlauf von Parametern (meist Serumkreatinin) erfasst und bei Überschreiten eines Grenzwerts ein Alarm ausgelöst wurde.

Das Problem der falschen Alarme ist nicht gelöst

Auf diese Weise ausgelöste Alarme sind zahlreich und führen daher rasch zu einer Missachtung der Alarme („E-Alert-Fatigue“). Bislang konnten selbst prominent publizierte KI-basierte Modelle das Problem der falschen Alarme nicht lösen. Das beste darin berichtete Ergebnis waren 2 falsche Alarme auf einen richtigen E‑Alert zu einer bevorstehenden AKI [28]. Ein rezent publiziertes KI-basiertes Alert-System berücksichtigte neben Serumkreatinin auch das Harnzeitvolumen sowie zusätzliche klinische Parameter u. a. Harnstoff, Leukozyten, Thrombozyten. Dieses DL-Modell konnte bei Intensivpatient*innen 88 % der AKI-Fälle mit einer Vorlaufzeit von mehr als 12 h vorhersagen. Von 6 Patient*innen, die mit einem hohen Risiko klassifiziert wurden, erlitten 5 auch tatsächlich eine AKI-Episode, hingegen trat AKI nur bei ca. 18 % der Patient*innen ohne vorhergesagtes Risiko auf [34]. Der Einsatz von KI könnte im Gegensatz zu den simplen Algorithmen zu gezielteren Alarmen führen. Die klinischen Effekte solcher auch in elektronische Patientenkurven (PDMS) integrierbarer E‑Alerts, die eine AKI bis zu 24 h vorhersagen könnten, wurden allerdings bislang noch nicht systematisch untersucht.

## KI-unterstütze Phänotypisierung

Eine AKI stellt als klinisches Syndrom mit losen Zusammenhängen von Symptomen [45] eine Sammeldiagnose für verschiedene Ausprägungen/Phänotypen der Nierenschädigung dar. Unter Einsatz von DL-Verfahren untersuchten verschiedene Analysen Methoden zur Einteilung einer bereits aufgetretenen AKI in Phänotypen [46]. Mithilfe dieser Phänotypisierung soll es möglich werden, prognostische Schlüsse zu ziehen oder die Therapie besser anpassen zu können [47] und auf diese Weise einen Schritt in Richtung personalisierte(re) Medizin zu machen. In diesem Kontext wurde versucht, mit ML-Modellen den Übergang von einem transienten zu einem persistierenden AKI vorherzusagen. In Bezug auf diese Fragestellung bei septischen Patient*innen erzielten alle untersuchten ML-Modelle („random forest“, „support vector machine“, „artificial neural network“, „gradient boosting“) nur mittelmäßige Vorhersagewerte (AUROC von ungefähr 0,75; [48]), während bei postoperativen Patient*innen höhere AUROC (0,837–0,856) von „support vector machine“, „decision trees“ und „gradient boosting“ erreicht wurden [49].

Mittels DL können verschiedene AKI-Phänotypen klassifiziert werden

Mittels DL ist es bereits möglich, verschiedene Phänotypen zu klassifizieren und unter kritisch kranken [50] und postoperativen Patient*innen [51] diejenigen mit einem höheren Mortalitätsrisiko zu identifizieren. Zusätzlich konnten septische Patient*innen in Gruppen mit einem niedrigen, moderaten und hohen Risiko für die Notwendigkeit einer Dialyse eingeteilt werden [52].

Bislang existieren jedoch erst wenige Arbeiten, die sich mit dem Nutzen der Phänotypisierung für die Therapie beschäftigten. Einzig eine Post-hoc-Analyse der VASST-Studie hat eine geringere Mortalität (27 % vs. 46 %) durch den Einsatz von Vasopressin (verglichen mit Noradrenalin) in einem spezifischen Phänotyp gezeigt. Dieser Phänotyp war durch eine bessere Nierenfunktion und höhere Raten von Sepsis, Vasopressoreinsatz und ARDS charakterisiert [53].

## Limitationen der KI

Auch wenn die Einsatzmöglichkeiten der KI-Technologien vielsprechend erscheinen, weisen sie derzeit noch eine Vielzahl an Schwächen auf, die nicht unerwähnt bleiben sollten. Die meisten derzeitigen Modelle beruhen nach wie vor primär auf Änderungen im Serumkreatinin und/oder in der Harnausscheidung [38]. Allerdings ist die Harnausscheidung nur wenig nierenspezifisch und das Serumkreatinin steigt erst mit Verzögerung an, wodurch diese Kriterien zumeist nicht in der Lage sind, frühe Stadien der AKI zu erkennen [54]. Zur Verbesserung der Frühdiagnostik wurden vor Kurzem subklinische Formen der AKI ohne Anstieg des Serumkreatinins, jedoch mit Schädigung des Nierenparenchyms und durch Anstieg von Biomarkern, wie z. B. NGAL, KIM‑1, TIMP2/IGFBP‑7, charakterisiert, postuliert [55].

Die Bestimmung dieser neuen innovativen Biomarker im Serum oder Urin ermöglicht ein frühzeitiges Erkennen subklinischer AKI [56]. Die KI-Modelle, die zusätzlich neue Biomarker, wie NGAL, zur AKI-Diagnostik berücksichtigen, wiesen eine deutlich höhere Genauigkeit auf [30]. Allerdings werden die neuen Biomarker nur selten in der klinischen Routine eingesetzt, wodurch sie (noch) kaum in größeren Datenbanken vorhanden sind. Die Dauer, bis Datensätze inklusive neuer Marker mit ausreichend großer Fallzahl zum Training von Algorithmen verfügbar sind, verursacht eine erhebliche Verzögerung in der Implementierung neuer Biomarkern in den ML-Modellen, wodurch der Fokus vorläufig auf Serumkreatinin und die Harnausscheidung gerichtet bleibt.

Falsche oder fehlende Werte in den Datenbanken können die Performance in der Trainingsphase negativ beeinflussen

Die KI-Algorithmen sind in der Performance abhängig von den Datenbanken, an denen sie trainiert werden. Während gewisse Datenbanken, wie die MIMIC-Datensets, bereits häufig verwendet wurden und in ihrer Qualität bekannt sind, sind viele Datensets zum Trainieren weniger oder nicht bekannt. Falsche oder fehlende Werte können die Performance in der Trainingsphase negativ beeinflussen [57]. Eine Metaanalyse [6] bestätigte das hohe Risiko für einen Bias auch bei Studien zu KI-Modellen. Darüber hinaus sind viele der großen Datenbanken durch ein gewisses Alter gekennzeichnet und rezente Entwicklungen in der Intensivmedizin sind noch nicht eingeflossen. Letztendlich können Datensets kein vollständiges Bild der Patient*innen abbilden, sondern sind nur in der Lage, sich durch Hinzufügen von (messbaren) Parametern diesem zu nähern.

## Kritischer Blick auf den derzeitigen KI-Einsatz

Auch wenn der Einsatz von KI ein vielversprechendes Tool in der Zukunft werden könnte, lohnt sich ein kritischer Blick auf den derzeitigen Einsatz. Durch die derzeitige Spezialisierung von KI-Modellen auf genau ein Krankheitsbild sind für ein vollumfängliches Erfassen von Patient*innen eine Vielzahl an KIs für unterschiedliche Syndrome nötig. Besonders bei kritisch kranken Patient*innen, die meist ein stark derangiertes Blutbild aufweisen, scheint der Einsatz von vielen verschiedenen ML-Algorithmen in der täglichen Routine kontraproduktiv, da der Masse an Warnungen und Empfehlungen nur mehr wenig Beachtung geschenkt werden und sie administrativ nur schwer zu bewältigen sein würde (E-Alert-Fatigue). Selbst unter Einsatz von DL blieb bislang die Rate von Fehlalarmen mit 2:1 immer noch sehr hoch [28].

Bei der Phänotypisierung fehlt bislang vielfach noch ein therapeutischer Mehrwert

Bei der Phänotypisierung fehlt bislang vielfach noch ein therapeutischer Mehrwert, der sich durch den Einsatz dieser Technologien ergibt und durch prospektive kontrollierte Studien bestätigt wurde. Ein weiterer Schwachpunkt ist die Tatsache, dass keinem der durch KI definierten Phänotypen bisher ein morphologisches Korrelat oder eine bestimmte Ätiologie zugeordnet werden konnte und in keiner der Analysen die Klassifizierung mit einer sinnvollen Intervention verbunden war. Aufgrund der Einteilung in Phänotypen ohne therapeutische Relevanz erscheinen diese Analysen derzeit mehr von akademischem als von klinischem Interesse zu sein.

Während bei manchen Algorithmen (noch) nachvollziehbar ist, wie ein bestimmtes Ergebnis Zustande kommt, sind besonders „artificial neural networks“ in der „hidden layer“ undurchsichtig, womit dem Kliniker die Nachvollziehbarkeit eines Befunds und damit die Rationale für eine (mögliche spezifische) Intervention fehlt. Die Modelle sind nur in der Lage, Veränderungen in den Werten zu interpretieren ohne das Zustandekommen dieser Parameter (z. B. Koinfektionen noch ohne Erregernachweis) berücksichtigen zu können. Nach Meinung der Autoren besteht das ärztliche Handeln jedoch viel mehr aus der Zusammenschau des klinischen Bilds und (wenn möglich) der Interaktion mit den Patient*innen als aus dem Behandeln von Laborwerten und Monitoren.

## Fazit für die Praxis

- Die nicht zuletzt durch die elektronischen Patientenkurven zunehmende Menge an Daten, die in großen Datenbanken zusammengefasst werden kann, ermöglicht es, die verschiedenen Machine-learning-Modelle schneller und mit aktuelleren Daten zu trainieren.
- Solange nicht neue Biomarker implementiert werden, bleibt das Problem der Limitierung auf Kreatinin und Harnausscheidung als insuffiziente Reflexion der Nierenfunktion bestehen.
- Nach Überwinden solcher und anderer Schwierigkeiten werden die verschiedenen ML-Modelle mit voranschreitender Digitalisierung Einzug in die klinische Praxis halten.
- Für ihren erfolgreichen Einsatz wird vor allem entscheidend werden, welches Vertrauen den immer komplexer werdenden ML-Modellen entgegengebracht wird.
- Als entscheidende Instanz wird der Mensch durch das Vereinen von messbaren mit nichtmessbaren Parametern jedoch unersetzlich bleiben. Die künstliche Intelligenz sollte und kann die natürliche Intelligenz der behandelnden Ärzt*innen nicht ersetzen.
